# Integrated Double-Sided Random Microlens Array Used for Laser Beam Homogenization

**DOI:** 10.3390/mi12060673

**Published:** 2021-06-09

**Authors:** Wei Yuan, Cheng Xu, Li Xue, Hui Pang, Axiu Cao, Yongqi Fu, Qiling Deng

**Affiliations:** 1 School of Physics, University of Electronic Science and Technology of China, Chengdu 610054, China; 201922120308@std.uestc.edu.cn (W.Y.); 201922120310@std.uestc.edu.cn (C.X.); xueli2553@163.com (L.X.); 2 Institute of Optics and Electronics, Chinese Academy of Sciences, Chengdu 610209, China; ph@ioe.ac.cn (H.P.); dengqiling@ioe.ac.cn (Q.D.)

**Keywords:** laser beam homogenization, microlens array, random, double exposure, integration

## Abstract

Double microlens arrays (MLAs) in series can be used to divide and superpose laser beam so as to achieve a homogenized spot. However, for laser beam homogenization with high coherence, the periodic lattice distribution in the homogenized spot will be generated due to the periodicity of the traditional MLA, which greatly reduces the uniformity of the homogenized spot. To solve this problem, a monolithic and highly integrated double-sided random microlens array (D-rMLA) is proposed for the purpose of achieving laser beam homogenization. The periodicity of the MLA is disturbed by the closely arranged microlens structures with random apertures. And the random speckle field is achieved to improve the uniformity of the homogenized spot by the superposition of the divided sub-beams. In addition, the double-sided exposure technique is proposed to prepare the rMLA on both sides of the same substrate with high precision alignment to form an integrated D-rMLA structure, which avoids the strict alignment problem in the installation process of traditional discrete MLAs. Then the laser beam homogenization experiments have been carried out by using the prepared D-rMLA structure. The laser beam homogenized spots of different wavelengths have been tested, including the wavelengths of 650 nm (R), 532 nm (G), and 405 nm (B). The experimental results show that the uniformity of the RGB homogenized spots is about 91%, 89%, and 90%. And the energy utilization rate is about 89%, 87%, 86%, respectively. Hence, the prepared structure has high laser beam homogenization ability and energy utilization rate, which is suitable for wide wavelength regime.

## 1. Introduction

Gaussian Laser beam has been widely used in the field of lighting [1], detection [2], and satellite communication [3]. However, for the applications of optical therapy, laser projection [4], and lithography [5], etc., it is necessary to homogenize Gaussian beam into a flat-topped beam. Aspheric lens group method [6,7,8], free-form lens method [9,10,11,12], diffracted optical elements (DOEs) [13,14,15], and microlens array (MLA) [16,17,18,19] are usually used to achieve laser beam homogenization. For the aspheric lens group method, the energy distribution of Gaussian beam is spatially modulated to form a uniform distribution in a specific position. The energy utilization rate and spot uniformity are both high up to 90%, which can be applied to lasers with high power [8]. In 2008, Oliker, V. [9] proposed a design method for the beam shaping of double free-form surface lens, which shapes the collimated incident laser beam into the collimated outgoing beam with the required energy distribution. In 2013, Feng, Z. proposed that the inhomogeneity of the laser beam can be eliminated by energy mesh dividing, expansion, and superposition [12]. This method has a high freedom of design which can effectively realize the high uniformity of the laser beam. However, the aspheric lens group is composed of two traditional aspheric lenses: concave lens and convex lens. Due to the large appearance size of the overall homogenization structure, the miniaturization, and integration of the optical system cannot be realized. For the free-form lens, the mesh division of lens surface is fine and each mesh surface will be designed separately, which brings high machining accuracy requirements and increase the machining difficulty. Diffractive optical elements (DOEs) with high integration can control the intensity distribution accurately with high diffraction efficiency. When the DOEs are used to homogenize the laser beam, the more the number of steps in the structure, the better the homogenization effect will, while the processing difficulty will increase as well. Meanwhile, the energy utilization rate, that is, diffraction efficiency will depend on the number of steps. For example, when the number of steps is two, four, eight, or sixteen, the energy utilization rate will be 40.5%, 81%, 94.9%, and 98.6%, respectively [20]. In addition, the DOEs operate in a very narrow wavelength band and is sensitive to the change of the wavelength, which limits the applicability of lasers with different wavelengths. The binary DOEs is always designed for a single wavelength. When the other wavelength is used to irradiate the DOEs, the central zero order strong intensity will be produced, which greatly reduces the uniformity of the homogenization spot. The method of MLA has the advantages of high energy utilization rate, small volume and high integration. In addition, it is not sensitive to the intensity distribution of incident light. The incident laser beam is divided into a series of sub-beams, which are superimposed on each other in the far field to eliminate the inhomogeneity between different sub-beams and form a homogenized spot [16,17]. The MLA is a refractive continuous surface structure with less stray light and it is suitable for beam homogenization of different lasers with a high energy utilization rate.

In recent years, a lot of related research work on the method of laser beam homogenization by means of using refractive MLA have been carried out. The imaging MLA (double periodic MLAs) beam integrator system was designed by Dickey F. M. for fiber injection to obtain uniform speckle pattern at the output end of the fiber [21] and the research results are applied in industry [22]. The higher the Fresnel number of the MLA, the sharper the edge of the homogenized spot will be, which is diffracted by the MLA. The uniformity of the homogenized spot is related to the number of sub-beams, which are divided by the MLA. However, periodic MLA is only suitable for the laser beam with poor coherence. In view of the laser beam with high coherence, the interference will occur between the sub-beams due to the periodic structure of the MLA, resulting in interference fringes in the obtained homogenized spots. Therefore, the periodic lattice phenomenon will appear, reducing the homogeneity of the spot greatly. In order to eliminate the influence of interference on the homogenized spot, researchers propose to employ the random phase plate (RPP) or the multifocal MLA to modulate the laser beam. Both of these methods disturb the coherence between sub-beams by modulating the phase, but they are implemented in different ways. The RPP is a typical optical birefringent material by randomly etching a specific depth of the unit structure on the surface. Thus, a random phase shift is introduced to modulate the polarization direction of the incident beam to disturb the coherence condition of the beam. In addition, a smooth uniform spot can be obtained [23]. The implementation scheme of multifocal MLA is the focus of this paper. Jin et al. [24] proposed to replace the first MLA with free-form MLA in the homogenizing system to improve the homogeneity in 2016. Each free-form surface in the MLA introduced an appropriate aberration in the wavefront to redistribute the irradiance of the beam. Compared to the traditional MLA homogenization system, the structure greatly reduced the diffraction effect and achieved a highly uniform beam profile. However, this method is only applicable to the laser beam with a larger diameter, since the aperture of the sub-lens is on the order of millimeter. To achieve the same uniformity for laser beams with small diameter, it is necessary to reduce the aperture of the MLA to the scale of micron and increase the number of MLA, which bringing in huge manufacturing difficulty of the MLA with free-form surface. Our research group have studied the laser beam homogenization using MLA. In 2016, Cao et al. [25] proposed a laser beam homogenization method using central off-axis random MLA. The periodicity of the MLA was broken by the off-axis quantity of the center to eliminate the periodic lattice effect at the target surface. However, the fabrication will become difficult due to the sharp change of the surface between the sub-lens units. Meanwhile, it is hard to install and align the two microlenses during the practical application. In 2020, Xue et al. [26] proposed a monolithic random MLA to homogenize the laser beam. In the process of beam homogenization, the coherence between sub-beams was completely broken, and the homogenized spot with high energy utilization rate was obtained. However, there is still a problem of adjustment and installation between the two plates when using the double MLA for beam homogenization.

Considering the problems and shortages of the previous research, a high integrated D-rMLA used to laser beam homogenization is proposed, which can be used to homogenize the laser beam with small diameter. D-rMLA is fabricated on a single substrate by double exposure and chemical etching technique. In the process of beam homogenization, the interference fringes between sub-beams are disturbed to obtain homogenized spots with high uniformity and energy utilization rate. In this paper, the method is used to carry out laser beam homogenization experiments and its feasibility is verified. The main arrangements of this paper are as follows: Section 2 describes the principle and simulation analysis of the beam homogenization based on the proposed D-rMLA, while Section 3 demonstrates the fabrication process of D-rMLA. Section 4 shows experimental results and verifies the validity of our method, and Section 5 is a summary of the whole paper.

## 2. Structure Design of D-rMLA

### 2.1. Principle of Beam Homogenization

**Figure 1:**
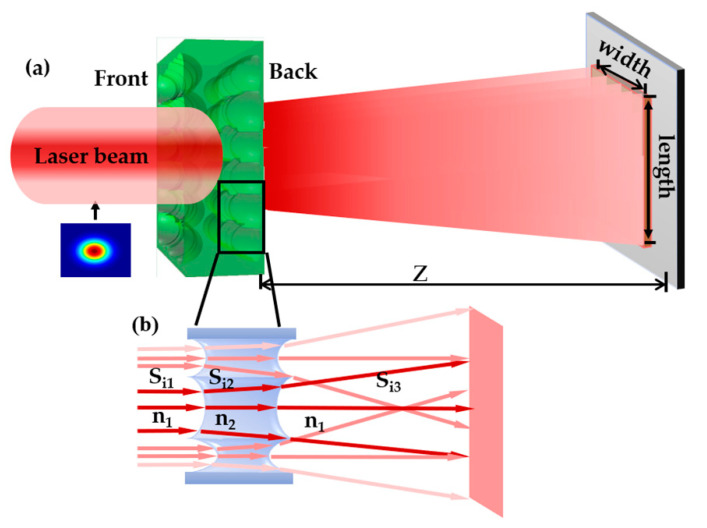
(**a**) Principle of laser beam homogenization; (**b**) a series of beam propagation paths.

The principle of laser beam homogenization based on D-rMLA is shown in Figure 1a. The collimated laser beam is incident on the front surface of the D-rMLA. Then the laser beam is divided into several sub-beams by the sub-lens in the D-rMLA. After that it is modulated by the structure of the back surface of the D-rMLA and emitted to the target surface. The distance between the back side of the D-rMLA and the target surface is Z. Due to the randomness of the aperture size, radius of curvature, and arrangement of each sub-lens of the D-rMLA, the interference conditions between the sub-beams are disturbed to improve the uniformity of the homogenized laser spot.

The beam propagation is shown in Figure 1b, and the difference of the transmission optical path of the sub-beams can be analyzed. The optical path calculation method is expressed by Equation (1).

(1) $$l_{i}=n_{1}\left(s_{i1}+s_{i2}\right)+n_{2}s_{i3}$$

where $n_{1}$ and $n_{2}$ are the refractive index of the different transmission mediums, and $s_{i1}$, $s_{i2}$ and $s_{i3}$ the transmission distance of one arbitrarily light path. Assume the refractive index is $n_{1}$ when the laser beam transmits in the air, and the refractive index is $n_{2}$ when the laser beam transmits in the glass medium. Because of the different structure parameters of different sub-lens, the different sub-beams transmit through different sub-lens units at the same location (such as the optical axis) will have different optical paths. Therefore, the different optical path differences $\Delta l_{n}$ will be generated between the sub-beams after the beam passing through the D-rMLA. The optical path difference can be expressed as Equation (2).

(2) $${\Delta \Phi }_{n}=\frac{2\pi }{\lambda }{\Delta l}_{n}$$

where $\Delta \Phi_{n}$ is the phase difference, and λ the wavelength of the incident laser beam. Due to the different $\Delta l_{n}$, As the different $\Delta \Phi_{n}$ will exist between the sub-beams after they are emitted through the D-rMLA. The conditions for coherent light are the same frequency, the parallel direction of vibration and the same phase or constant phase difference. The conditions for the coherence of the incident beam are disturbed due to the random phase difference. Thus, a spot with uniform energy distribution on the target surface can be obtained.

### 2.2. Simulation

**Figure 2:**
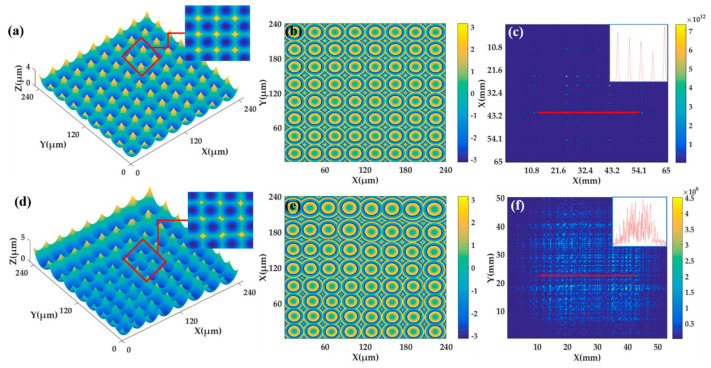
The simulation of periodic MLA and D-rMLA: (**a**) the three-dimensional (3D) structure of the MLA; (**b**) the phase of periodic MLA; (**c**) the obtained homogenized spot; (**d**) The 3D structure of the rMLA; (**e**) the phase distribution of the rMLA; (**f**) the homogenized spot of D-rMLA.

The effects of beam homogenization of double periodic MLA and D-rMLA were simulated and calculated by means of the numerical analysis software of Matlab (Version 7.1, MathWorks, Natick, MA, USA). The beam homogenization ability of different structures of MLA is compared. The aperture, radius of curvature and array number of the sub-lens in the periodic MLA are designed as 30 μm × 30 μm, 28 μm, and 8 × 8, respectively. Diffraction distance is designed as 20 mm. According to the design parameters, the surface function Formula (3) is used to establish the MLA model, so that each microlens can be expressed numerically. Meanwhile, the transfer function of the MLA can be calculated by Formulas (4) and (5). The phase component of the MLA can be decomposed from the transfer function to calculate the phase distribution of each microlens. According to Fresnel diffraction theory, the homogenized spot with diffraction distance of 20 mm can be calculated. The three-dimensional (3D) structure of the MLA is shown in Figure 2a. Figure 2b is the phase distribution of the periodic MLA. The obtained homogenized spot is shown in Figure 2c. It can be seen that the homogenized spot of the periodic MLA is consisted of periodic dot matrix.

(3) $$\mathrm{sag}\left(x_{i},y_{i}\right)=\frac{C_{i}r_{i}{}^{2}}{1+\sqrt{1-\left(\kappa +1\right)C_{i}{}^{2}r_{i}{}^{2}}},\;r_{i}=\sqrt{x_{i}{}^{2}+y_{i}{}^{2}}$$

(4) $$\Phi_{i}=\frac{2\pi }{\lambda }\left(n-1\right){\mathrm{sag}}_{i}$$

(5) $$t_{i}\left(x_{i},y_{i}\right)=\exp({\mathrm{ik}\Delta }_{0})\exp[{i\Phi }_{i}]$$

In order to compare the periodic MLAs, the side length of quadrilateral apertures of the sub-lenses in D-rMLA are designed to vary randomly from 25 μm to 45 μm, that is (30 − 5 × rand (0, 1), 30 + 15 × rand (0, 1)) μm. The array number and the average radius of curvature of the D-rMLA is 8 × 8 and 25 μm, respectively. The 3D structure of the rMLA is shown in Figure 2d. Figure 2e is the phase distribution of the rMLA. The homogenized spot of the laser beam transmitting through the D-rMLA at the distance of 20 mm is shown in Figure 2f. Simulation results show that the D-rMLA can eliminate the interference phenomenon caused by the periodicity of the traditional MLA with better homogenization effect. The homogenized spot of periodic MLA presents periodic lattice distribution, and the energy is only strong diffraction orders. The energy of the homogenized spot of the D-rMLA is more discrete and uniform.

## 3. Fabrication of D-rMLA

The fabrication methods of MLA mainly include thermal reflow technique [27], laser direct writing (LDW) [28], 3D printing [29], and gray-scale lithography [30]. Thermal reflow technique is an efficient way to produce MLA by preparing an array of photoresist polymer cylinders regularly distributed on a substrate and melting the cylinders into a hemispherical shape [27]. However, the filling factor of this preparation method cannot reach 100%. When it is used in beam homogenization, some lights will not be modulated resulting in a central bright spot, which reduces the quality of beam homogenization. LDW is to use the laser beam with variable intensity to expose the resist material on the surface of the substrate. After development, the required relief profile will be formed on the surface of the resist layer [28]. Two-photon polymerization (TPP) 3D printing can also achieve the fabrication of MLA with hundred-nanometer-scale or sub-micrometer-scale resolution [29]. Nevertheless, the LDW and TPP 3D printing techniques are based on point-by-point structural modification and require a long fabrication time for producing large-sized components. Thus, it is time-consuming and costly to fabricate large-scale components. Furthermore, gray-scale lithography is proposed to fabricate the MLA [30]. In the process of exposure, MLA with continuous surface is fabricated by moving the mask dynamically. The fabrication method is flexible and does not need expensive cost. However, for the fabrication of MLA with small apertures, the number of sampling and quantizing is limited, which will reduce the surface quality of the profile.

Although a lot of research work about the fabrication of MLA has been demonstrated, only few multi-focus MLA which consist of lens lets of different focal lengths were presented in literature. In 2012, Ferraro et al. group proposed the pyroelectric continuous printing method to fabrication graded size MLA, which has different focal lengths [31,32]. The technology limitation strongly depend on the viscosity of the polymeric material. The higher the viscosity, the higher the diameter of the lenses will be. It is difficult to achieve high fill factor. Moreover, the fabrication method which combining lithography and chemical etching has been proposed to prepare rMLA in the previous study [26]. It can realize the fabrication of randomly distributed MLA with small aperture and multi focal length at low cost. The pattern on the mask was transferred to the glass substrate by lithography technology, then the random microlens structure was etched on the glass substrate through the chemical etching method. The double-sided exposure technology is proposed on the basis of the fabrication of rMLA to realize the high precision alignment of the double-sided sub-lens unit structure on both sides of the substrate in this paper to obtain the integrated D-rMLA.

**Figure 3:**
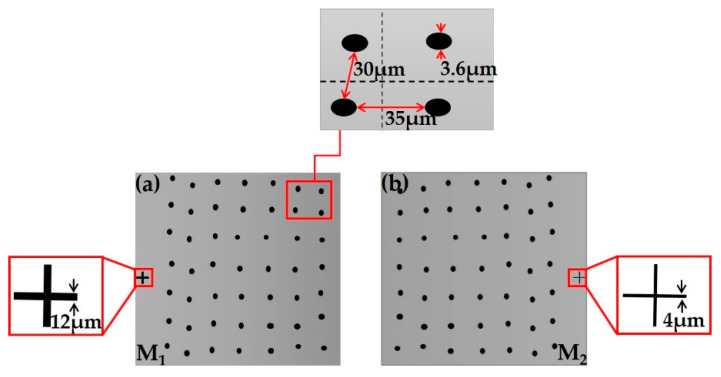
Part patterns of mask: (**a**) the first mask M_1_; (**b**) the second mask M_2_.

In order to achieve the spatial alignment of the microstructures on both sides of the glass substrate, it is necessary to prepare two masks, M_1_ and M_2_, with random hole arrays distributed in mirror image, as shown in Figure 3. The diameter of the hole is 3.6 μm. The interval of the central position in the holes vary from 25 μm to 45 μm, that is (30 − 5 × rand (0, 1), 30 + 15 × rand (0, 1)) μm. Moreover, an alignment mark, which is a crosshair, was designed on each mask to align the two sides of the sub-lens at the process of exposure. The crosshair on the mask M_1_ for the first exposure is a wider crosshair with a line width of 12 μm. Furthermore, the crosshair on the mask M_2_ for the second exposure is a thinner crosshair with a line width of 4 μm.

**Figure 4:**
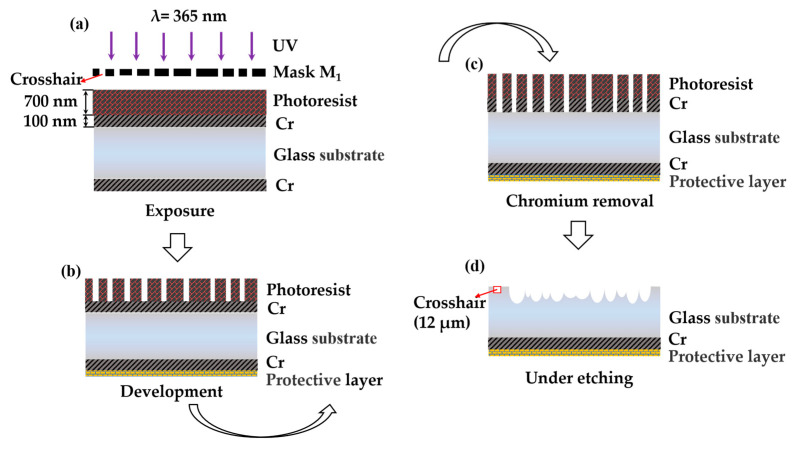
Fabrication process of single-sided rMLA: (**a**) exposure; (**b**) development; (**c**) chromium removal; (**d**) etching.

Firstly, the single-sided rMLA is prepared on one side of the substrate. The process flow is shown in Figure 4, including exposure (using M_1_), development, dechroming, and etching. At this time, the crosshair with a line width of 12 μm is etched on the edge region which is near the structure of rMLA.

Secondly, another rMLA structure is fabricated on the other side of the substrate. The preparation method is described in detail in the paragraph below.

**Figure 5:**
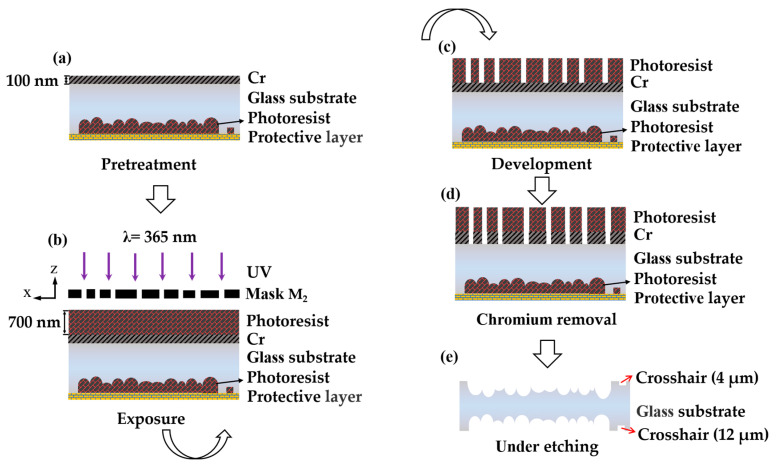
Fabrication process of D-rMLA: (**a**) pretreatment; (**b**) double sided exposure; (**c**) development; (**d**) chromium remove; (**e**) etching of the D-rMLA.

The first step is the pretreatment of the substrate, including the protection of the prepared single-sided rMLA and the preparation of the mask layer namely the chromium film using chemical etching, as shown in Figure 5a. The fabricated rMLA is filled with photoresist and pasted with acid-fast as protective layer to protect the fabricated structure from corrosion by HF. At the same time, a chromium film with a thickness of 100 nm is plated on the polished surface of the substrate, which is used as a masking layer for chemical etching.

**Figure 6:**
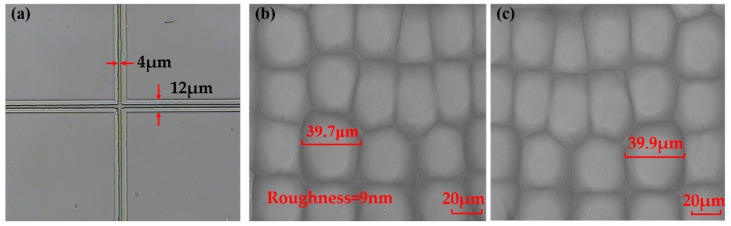
Microscopic image of the D-rMLA: (**a**) crosshair alignment picture; (**b**) MLA micrograph of the first surface; (**c**) MLA micrograph of the second surface.

The second step is photoresist coating and exposure. The photoresist of the type is AZ MIR—703 Photoresist (14 cp) (AZ Electronic materials, Somerville, MA, USA) which was spin-coated on the glass substrate with chromium film at the speed of 4000 r/min with time of 30 s. The thickness of the photoresist was about 700 nm. In order to ensure that there is no rotation error between both sides of the substrate rMLAs after etching, alignment marks were used to align during the exposure process. The crosshair structure M_1_ which had been etched on the substrate was aligned with the crosshair on the mask M_2_. In the process of exposure, the alignment marks on the mask M_2_ were captured with the microscope. Then the prepared rMLA was placed on the wafer supporting with the rMLA structure face down and the photoresist face up, as shown in Figure 5b. By translating or rotating the wafer supporting, the crosshair with 4 μm line width on the mask was nested in the crosshair with 12 μm line width which had been prepared on the back of the substrate, as shown in Figure 6a, so as to achieve strict alignment of the rMLA on both sides. Then lock the wafer supporting for exposure. The exposure power density was set as 3 mW/cm^2^ with the center wavelength of 365 nm, and the exposure time was 20 s. In the process of alignment exposure, the alignment deviation of the crosshair mark is with ±1 μm.

The third step is development. By the development with developer of AZ 300MIF DEVELOPER (AZ Electronic Materials, Somerville, MA, USA) for 35 s, the microporous structure on the mask was transferred to the photoresist, as shown in Figure 5c.

The fourth step is chromium removing. The developed substrate is put into the chromium-removing solution for about 40 s to transfer the microporous structure on the photoresist to the chromium film, as shown in Figure 5d.

**Figure 7:**
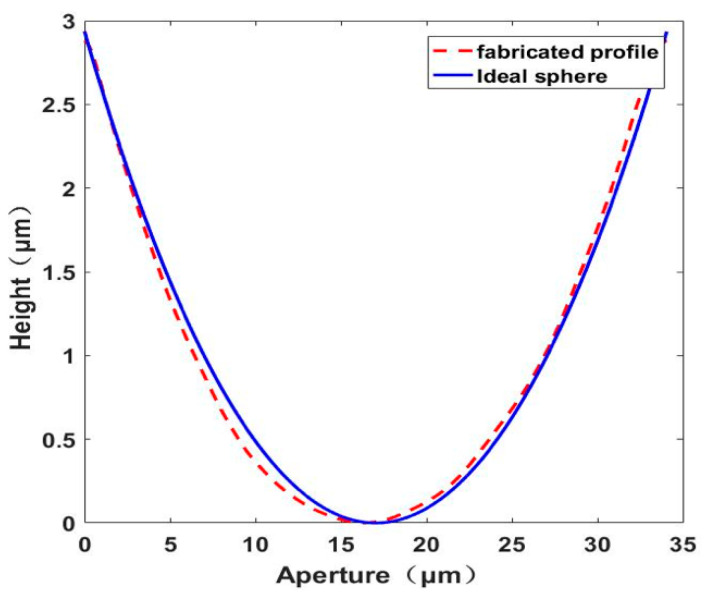
Profile Comparison between the fabricated microlens with ideal sphere.

The last step is etching. The protected substrate is etched in etching solution (H_2_O:HF:HNO_3_ = 5:2:2). In the etching process, the glass substrate is taken out every 3 min and put into the ultrasonic oscillator to clean the chemical reaction residue. The rMLA on both sides of the substrate has the same distribution of diameters and sag heights after the same etching time of 30 min, as shown in Figure 5e. The microscope pattern of the rMLA can be obtained after etching. The structure of the front and back sides was mirror symmetrical, whose filling factors are 100%, as shown in Figure 6b,c. Then the roughness and apertures of the prepared D-rMLA were measured by step profilometer (Stylus Profiler System, Dektak XT, Bruker, Karlsruhe, Germany). The resolution of the step profilometer is 1 Å. The roughness is about 9 nm in average, and the apertures vary form 25 μm to 45 μm, which is consistent with the designed parameters of the masks. The profile of the sub-lens was also fitted, as shown in Figure 7. The profile of the fabricated sub-lens unit almost coincides with the ideal sphere, showing high profile accuracy. The depths of the sub-lenses vary from 2 μm to 4 μm.

## 4. D-rMLA Testing

The experimental optical paths are constructed on the basis of the fabricated D-rMLA to test the laser beam homogenization and verify the applicability of the lasers with different wavelengths.

Firstly, the divergence angles of the homogenized spots generated by the D-rMLA of different wavelengths are measured. In the experiment, the laser beams with wavelengths of 650 nm (R), 532 nm (G), and 405 nm (B) were irradiated to the D-rMLA, respectively. The powers of the sources of RGB laser radiation are 100 mW, 100 mW, and 30 mW, respectively. The transverse mode of the RGB laser radiation is near TEM_00_, which has high coherence. By the phase modulation of the D-rMLA the homogenized spots were obtained. The propagation distance *Z* between the Charge Coupled Device (CCD, the resolution of 4896 × 3248 and pixel size of 7.4 μm × 7.4 μm) and D-rMLA was measured as 20 mm, and the incident spot size T_0_ was 4 mm. Therefore, if the size of the homogenized spot T can be measured, the half divergence angle θ of the homogenized spot at different wavelengths can be calculated by Equation (6).

(6) $$\theta =\arctan\frac{T-T_{0}}{2Z}$$

The homogenized spots of different wavelengths were captured by CCD, and the sizes of the homogenized spots were calculated by numerical analysis software MATLAB. The energy of the homogenized spots attenuates to 1/e^2^ is counted as T, which can be obtained by multiplying the number of pixels of the spot by the pixel size. For the laser beam with wavelength of 650 nm (R), the pixel number of homogenized spots is accounted as 2093, and the T and the full divergence angle were calculated as 15.5 mm and 32°, respectively. For the laser beam with wavelength of 532 nm (G), the pixel number was 1929, and the T and the full divergence angle were calculated as 14.3 mm and 29°. For the laser beam with wavelength of 405 nm (B), the pixel number was 1799, and the T and the full divergence angle were calculated as 13.3 mm and 26°. For the fabricated D-rMLA, the central zero order strong intensity will not be produced by the incident beams with different wavelengths. The divergence angle of the homogenized spot will change when different wavelength beams are incident. According to the grating equation (*d × sin θ = λ*), the longer the wavelength is, the larger the diffraction angle is.

**Figure 8:**
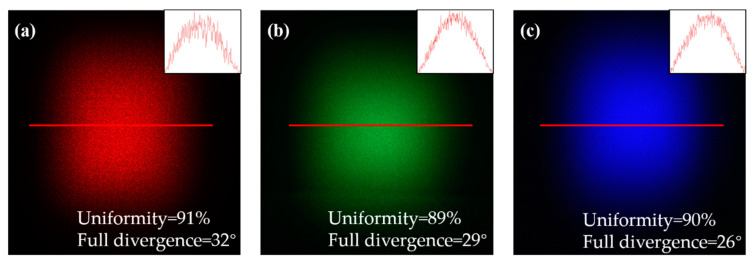
Homogenized spots of the laser with different wavelengths: (**a**) 650 nm; (**b**) 532 nm; (**c**) 405 nm.

Secondly, the uniformity of homogenized spots was tested. The homogenized spots of the laser with different wavelengths were captured after laser homogenization, as shown in Figure 8. The uniformity was calculated by Equation (7). The uniformity of 650 nm (R), 532 nm (G), and 405 nm (B) was 91%, 89%, and 90%, respectively. It can be seen that the uniformity of the homogenized spots for different wavelengths is very high with a little difference. Comparing the homogenization results of simulation and experiment, it is found that the homogenized spot is no longer composed of strong diffraction orders, the energy of the homogenized spot of the D-rMLA is more discrete and uniform. The experimental and simulation results are consistent.

Finally, the energy utilization rate is tested. The power of the incident and outgoing laser beams is measured by the power meter (accuracy of 1 μW) and the energy utilization rate is calculated by Equation (8). The power P_in_ of the incident beam of 650 nm (R) is 0.98 mW, and the outgoing power P_out_ is 0.87 mW. The energy utilization rate is calculated as 89%. The power P_in_ of the incident beam of 532 nm (G) is 2.05 mW and the outgoing power P_out_ is 1.8 mW. The energy utilization rate was calculated as 87%. The power P_in_ of the incident beam of 405 nm (B) is 4 mW, and the outgoing power P_out_ is 3.44 mW. The energy utilization rate is calculated as 86%. Therefore, it can be concluded that the structure of D-rMLA has a high energy utilization rate. The energy loss mainly occurs in the reflection of the substrate surface, the internal absorption or scattering because of the refraction type of the fabricated D-rMLA with a continuous surface. The above three types of the energy loss are very small, so the energy utilization rate of the prepared structure is very high.

(7) $$\mathrm{RMSE}=\sqrt{\overset{N}{\underset{j}{\sum }}{\left(I_{j}-\bar{I}\right)}^{2}/N}$$

(8) $$\eta =\frac{P_{\mathrm{in}}}{P_{\mathrm{out}}}\times 100\%$$

## 5. Conclusions

This paper has proposed an integrated D-rMLA fabricated by double-sided exposure technique for laser beam homogenization. The experimental results show that the uniformity of the homogenized spot for the laser beams with wavelengths of 650 nm (R), 532 nm (G), and 405 nm (B) are 91%, 89%, and 90%, respectively. The energy utilization rate is 89%, 87%, and 86%, respectively. It is verified that the integrated D-rMLA can break the interference lattice phenomenon brought by the periodic MLA and improve the homogenization quality. Compared with the traditional double MLAs in series, the homogenizer greatly improves the system integration and can be used in laser projection, laser backlighting, and other fields.

## Data Availability

Data will be provided on request through the corresponding author (Axiu Cao) of this article.
